# Mortality during and after specialist alcohol and other drug treatment: Variation in rates according to principal drug of concern and treatment modality

**DOI:** 10.1111/dar.13669

**Published:** 2023-04-25

**Authors:** Alys Havard, Nicola Jones, Chrianna Bharat, Natasa Gisev, Sallie‐Anne Pearson, Anthony Shakeshaft, Michael Farrell, Louisa Degenhardt

**Affiliations:** ^1^ National Drug and Alcohol Research Centre, UNSW Sydney Sydney Australia; ^2^ Centre for Big Data Research in Health, UNSW Sydney Sydney Australia

**Keywords:** data linkage, drug, mortality, treatment

## Abstract

**Introduction:**

For people accessing treatment for problems with drugs other than opioids, little is known about the relationship between treatment and mortality risk, nor how mortality risk varies across treatment modalities. We addressed these evidence gaps by determining mortality rates during and after treatment for people accessing a range of treatment modalities for several drugs of concern.

**Methods:**

We conducted a cohort study using linked data on publicly funded specialist alcohol or other drug treatment service use and mortality for people receiving treatment in New South Wales between January 2012 and December 2018. We calculated and compared during‐treatment and post‐treatment crude mortality rates and age‐ and sex‐standardised mortality rates, separately for each principal drug of concern and modality.

**Results:**

Over the study period, 45,026 people accessed treatment for problems with alcohol, 26,407 for amphetamine‐type stimulants, 23,047 for cannabinoids and 21,556 for opioids. People treated for alcohol or opioid problems had higher crude mortality rates (1.48, 1.91, 1.09 per 100 person years, respectively) than those with problems with amphetamine‐type stimulants or cannabinoids (0.46, 0.30 per 100 person years, respectively). Mortality rates differed according to treatment status and modality only among people with alcohol or opioid problems.

**Discussion and Conclusions:**

The observed variation in mortality rates indicates there is scope to reduce mortality among people accessing treatment with alcohol or opioid problems. Future research on mortality among people accessing drug and alcohol treatment should account for the variation in mortality by drug of concern and treatment modality.

**Key Points**

- This is the first comprehensive study of mortality among people accessing different specialist alcohol and other drug treatment modalities for several drugs of concern.
- Mortality rates were higher among people with alcohol or opioid problems, relative to other drugs.
- Among people with alcohol or opioid problems, mortality rates were lower during treatment than after treatment for some treatment modalities.
- During‐treatment and post‐treatment mortality rates varied by treatment modality among people with alcohol or opioid problems.
- Findings indicate there is scope to reduce mortality among people accessing treatment with alcohol or opioid problems.

## 1 INTRODUCTION

People with alcohol or other drugs problems are more likely to die prematurely than their peers in the general population. Compared to age and sex peers, mortality risk is 3.6 times higher among people with alcohol use disorder [1], 6.8 times higher among people with regular or dependent amphetamine use [2] and 14.7 times higher among people with regular or dependent opioid use [3]. Published data are insufficient to determine whether mortality is elevated among cannabis users [4].

There is evidence that engaging in drug and alcohol treatment is associated with reduced mortality, but this evidence primarily relates to treatment for opioid dependence. Systematic reviews of studies focused on people receiving opioid agonist treatment (OAT) found lower rates of all‐cause and cause‐specific mortality during treatment, compared to out‐of‐treatment [3, 5]. Individual cohort studies have found reduced in‐treatment mortality rates for people accessing other treatments for opioid dependence, including withdrawal management [6, 7], psychosocial treatments [6] and residential treatments [8]. There has been limited investigation of the relationship between treatment and mortality for drugs other than opioids. To our knowledge, this has been examined for cannabis only, with a single study finding no difference in mortality rates during and after treatment (various modalities combined) [9]. In a study among people accessing treatments for a range of drugs in Victoria, Australia, mortality during treatment was higher than in the 2 years after treatment [10]. The authors note the inconsistency between this finding and existing evidence, however, hypothesising it is due to the aggregation of a diverse range of treatment modalities and drugs. Whether engagement in treatment is associated with reduced mortality among people with problematic use of drugs other than opioids, and whether this varies across different treatment modalities, is largely unknown.

Even for treatment modalities with a demonstrated association with reduced mortality relative to periods out of treatment, there remains excess risk of mortality relative to the general population. Specifically, mortality rates during and after OAT exceed mortality rates among the general population [11]. Excess mortality risks have also been found among people accessing other treatment modalities for other drugs of concern [1, 10, 12, 13]. This suggests that there are further opportunities to reduce the risk of premature mortality among people accessing drug and alcohol treatment. Designing service enhancements that optimise client outcomes while making best use of limited resources requires an understanding of the treatment populations most at risk of premature mortality, including whether the risk during treatment and after treatment varies according to treatment modality. This question has been somewhat addressed for opioid dependence treatments, with OAT associated with lower during‐treatment mortality risk than community psychological support [8], and similar during‐treatment risk as residential treatments [8] and withdrawal management [7]. In contrast, mortality risk in the post‐treatment period is higher among those discharged from residential treatment and OAT than those discharged from psychological support [8].

To our knowledge, there has been no prior investigation of how mortality rates during treatment vary across treatment modalities for drugs other than opioids. There has, however, been some examination of how post‐treatment mortality rates vary. The above‐mentioned Victorian study found the highest rates of mortality in the 2 years following residential withdrawal management, and lowest mortality in the year following counselling [10]. A similar study in Texas found significantly higher drug‐related mortality rates in the year following discharge from residential treatments, withdrawal management and OAT compared to those discharged from outpatient treatment [14]. Neither of these studies, however, reported mortality rates separately by principal drug of concern (PDoC), which is associated with much variation in mortality risk.

Our objective was to address these evidence gaps for people accessing a range of specialist drug and alcohol treatment modalities across a range of PDoCs. Specifically, we compared mortality rates during and after treatment, separately for each PDoC and treatment modality. We also examined whether mortality rates during and after treatment vary according to treatment modality, separately for each PDoC.

## 2 METHODS

This cohort study was based on linked data on health service use and mortality for people receiving publicly funded specialist alcohol or other drug treatment in New South Wales (NSW), Australia's most populous state.

### 2.1 Data sources

The NSW Minimum Data Set for Drug and Alcohol Treatment Services (MDS DATS) records all services provided by government and non‐government drug and alcohol agencies receiving NSW Ministry of Health funding for providing specialist drug and/or alcohol and/or gambling services. The PDoC for each treatment episode is coded according to the Australian Standard Classification of Drugs of Concern [15, 16]. The MDS DATS extract for this study included only closed treatment episodes.

The NSW Admitted Patient Data Collection records admissions to all NSW hospitals. Diagnoses are coded according to the International Statistical Classification of Diseases and Related Health Problems, Tenth Revision, Australian Modification [17], procedures are coded according to the Australian Classification of Health Interventions [18] and admissions as a whole are classified according to Australian‐Refined Diagnostic Related Groups [19].

Death records from the NSW Registry of Births, Deaths and Marriages records the date of every death registered in NSW.

Records from these collections were linked for all people with a MDS DATS record with a treatment exit date between 1 January 2012 and 31 March 2019 or a record in the non‐admitted patient (NAP) data collection with a service start date between 1 July 2015 and 30 September 2018, where the service provided was coded as ‘20.52: Addiction Medicine’ or ‘40.30: Alcohol and Other Drugs’ [20]. The NAP data collection includes data on outpatient services provided by the NSW public hospital system. Although people receiving NAP services were included in our cohort, because the PDoC is not specified, we did not use NAP treatment records in our analyses.

The linkage was performed by the NSW Centre for Health Record Linkage using personal identifiers. As MDS DATS records from before July 2015 did not contain full personal identifiers, linkage of these early records used a statistical linkage key based on partial identifiers. The rate of false positive links, based on the Centre for Health Record Linkage's review of a random 1000 person IDs, was 0.5%.

### 2.2 Study cohort

We identified people who, in the period 1 January 2012 and 31 December 2018, had at least one MDS DATS record or a hospital admission for withdrawal management for alcohol use disorder, as recorded in the Admitted Patient Data Collection. We excluded: people who had no treatment episodes with one of the four nominated PDoCs of interest (see Section 2.5); those seeking treatment for gambling disorder or for another person's substance use; people with inconsistent information across different data sources; and people for whom age and sex could not be determined.

We conducted a sensitivity analysis by restricting our cohort to people entering treatment from 1 July 2015 onwards (sensitivity analysis 1) so as to examine the impact of potentially reduced accuracy of links involving early MDS DATS records.

### 2.3 In‐treatment and post‐treatment periods

For people already in treatment at the beginning of the study period, we began follow up on 1 January 2012. For people commencing their first treatment episode after 1 January 2012, we began follow‐up on their treatment start date. We followed people from this date through to 31 December 2018 (or until the date of death, if this occurred earlier). We used the treatment entry and exit dates recorded in the MDS DATS and the admission and discharge dates in the admitted patient data collection to group person‐time into in‐treatment and post‐treatment periods and subdivided these periods according to the treatment modality accessed and the PDoC.

We excluded post‐treatment days occurring after 31 March 2018. This is because we had data on closed treatment episodes only and people may have commenced a new treatment episode that was still open when the data were extracted (31 March 2019). We considered this risk to be highest for the last 12 months of data availability as the MDS DATS data collection rules specify (with some exceptions) that a treatment episode should be closed (and a new episode record created) when it reaches the maximum length of 12 months [21].

### 2.4 Treatment modality

We focused on nine modalities of specialist alcohol and other drug treatment: assessment only, counselling, involuntary drug and alcohol treatment (IDAT), OAT, outpatient consultation, rehabilitation, residential rehabilitation, support and case management, and withdrawal management. We identified these modalities by combining information from the MDS DATS field of ‘main service provided’ with information regarding the setting in which the service was delivered, as defined in Table S1, Supporting Information.

We also used hospital records to identify episodes of withdrawal management for alcohol use disorder based on an International Statistical Classification of Diseases and Related Health Problems, Tenth Revision, Australian Modification diagnosis code related to problematic use of alcohol, as well as a relevant Australian Classification of Health Interventions code in the primary procedure field or a relevant Australian‐Refined Diagnostic Related Groups code (Table S2).

We observed that 8.8% of treatment episodes overlapped with at least one other episode, suggesting that some people received multiple treatments simultaneously. For the purposes of the analyses, we considered each treatment modality independently. We also conducted a sensitivity analysis using data related to single service episodes only (sensitivity analysis 2).

### 2.5 Principal drug of concern

We defined four broad PDoC categories: alcohol, amphetamine‐type stimulants, cannabinoids and opioids. See Table S3, for the drugs comprising each of these categories.

### 2.6 Mortality rates

We attributed deaths to an in‐treatment period if they occurred during the episode or on the day after treatment exit. We included deaths on the day after treatment exit to account for the likely scenario in which an individual dies and stops presenting for treatment, hence the treatment provider closes the treatment episode and records the client was last seen as the treatment end date. While our main analysis assumed a one‐day grace period, sensitivity analysis 3 examined whether alternative grace periods (0 days and 7 days) returned different results.

For the treatment modality of assessment only, where the episode represents a single treatment session, we attributed all person‐time and deaths on the date of assessment or thereafter to the post‐treatment period. Accordingly, our analyses for assessment only included no in‐treatment person‐time.

For each in‐treatment and post‐treatment period of interest, we computed crude mortality rates (CMR) as the total number of deaths occurring during the period divided by the total person‐time in that period. We present this as a rate of death per 100 person‐years. As CMRs are assumed to follow the Poisson distribution, we calculated 95% confidence intervals (CI) for the CMRs based on a Poisson distribution [22]. We also computed direct age‐ and sex‐ standardised mortality rates (ASR) for each period of interest. To calculate ASRs, we calculated the CMRs for each 10‐year age and sex stratum in our cohorts, and applied the age‐, and sex‐ specific weights from 2015 NSW population estimates [23]. We calculated the 95% CIs for the ASRs using the normal approximation [24].

We considered CMRs/ASRs for different time periods or treatment modalities to be statistically significantly different if the 95% CIs around the rates did not overlap. In the case of overlapping confidence intervals, noting that this does not necessarily indicate the difference between the estimates is not significant [25], we based our interpretation on inspection of the CMR/ASR estimates and the uncertainty surrounding those estimates.

Sample size calculations assuming a CMR of 0.89 deaths per 100 person years (the CMR among people who accessed OAT in NSW [11]) indicated that estimating the true mortality rate within ±0.5 deaths per 100 person years would require 1360 person years of observation. We therefore defined a reporting threshold such that we reported CMRs only for treatment modalities with at least 1360 person years of observation available for the in‐treatment and/or post‐treatment period. We allowed an exception to this reporting threshold for IDAT where alcohol was nominated as the PDoC given the added importance of understanding mortality risk in this group and the lack of existing evidence.

Ethics approval to conduct this study was granted by the UNSW Human Research Ethics Committee (ref: HC210443).

## 3 RESULTS

A total of 102,256 people met our cohort inclusion criteria (Figure S1 quantifies the exclusions). Over the study period, 45,026 people accessed treatment for alcohol problems, 26,407 people for amphetamine‐type stimulants, 23,047 for cannabinoids and 21,556 for opioids.

The CMR among people accessing treatment for alcohol problems was 1.48 per 100 person years (PY) [95% CI 1.41–1.55] (Table 1), while the ASR was 1.64 per 100 PY [95% CI 1.54–1.73]. The CMR among people accessing treatment for opioid problems was significantly lower (1.09 [1.01–1.17]), but after age‐ and sex‐ standardisation, the mortality rate (ASR 1.56 [1.31–1.81]) was similar to that for alcohol. People accessing treatment for problems with amphetamine‐type stimulants or cannabinoids had significantly lower CMRs than people accessing treatment for alcohol or opioid problems. They also had significantly lower ASRs than people treated for alcohol problems.

**TABLE 1:** Crude mortality rates and age‐and sex‐standardised mortality rates for people accessing alcohol and/or drug treatment in New South Wales, 2012–2018.

Principal drug of concern	Person years	Deaths	CMR per 100 PY [95% CI]	ASR per 100 PY [95% CI]
Alcohol	117,766	1747	1.48 [1.41–1.55]	1.64 [1.54–1.73]
Amphetamine‐type stimulants	53,242	246	0.46 [0.41–0.52]	1.03 [0.55–1.51]
Cannabinoids	51,223	153	0.30 [0.25–0.35]	0.68 [0.31–1.05]
Opioids	70,093	763	1.09 [1.01–1.17]	1.56 [1.31–1.81]

Abbreviations: ASR, age‐ and sex‐standardised mortality rate; CI, confidence interval; CMR, crude mortality rate; PY, person years.

The number and characteristics of people accessing each treatment modality are reported separately by PDoC in Tables S4a–d. Inspection of these descriptive data suggests substantial variation between treatment modalities in stable accommodation, injecting drug use history and hospitalisation history. Mortality rates during and after treatment are reported in the sections that follow, separately by PDoC and treatment modality.

### 3.1 Alcohol

Among people with alcohol problems, mortality rates during and after treatment were computed for seven of the nine treatment modalities (see Table 2). In‐treatment and post‐treatment person‐time for OAT and rehabilitation fell below the reporting threshold.

**TABLE 2:** Crude mortality rates and age‐ and sex‐standardised mortality rates by treatment modality for people accessing treatment with alcohol as the principal drug of concern in New South Wales, 2012–2018.

	Person years	Deaths	CMR per 100 PY [95% CI]	ASR per 100 PY [95%CI]
Assessment only				
In treatment	N/A	N/A	N/A	N/A
Post treatment	8936	75	0.84 [0.66–1.05]	0.94 [0.67–1.21]
Counselling				
In treatment	7325	32	0.44 [0.30–0.62]	0.39 [0.23–0.54]
Post treatment	39,968	349	0.87 [0.78–0.97]	0.88 [0.77–0.99]
Involuntary drug and alcohol treatment				
In treatment	43	0	0.00 [0.00–8.54]	^a^
Post treatment	82	7	8.56 [3.44–17.64]	^a^
Outpatient consultation				
In treatment	693	6	0.87 [0.32–1.89]	^a^
Post treatment	6311	137	2.16 [1.82–2.56]	1.90 [1.52–2.27]
Residential rehabilitation				
In treatment	1229	0	0.00 [0.00–0.30]	^a^
Post treatment	7609	43	0.57 [0.41–0.76]	0.65 [0.40–0.91]
Support and case management				
In treatment	1494	11	0.74 [0.37–1.32]	^a^
Post treatment	9270	89	0.96 [0.77–1.18]	1.06 [0.80–1.32]
Withdrawal management				
In treatment	1128	10	0.89 [0.42–1.63]	^a^
Post treatment	27,101	372	1.37 [1.24–1.52]	1.22 [1.06–1.38]

Abbreviations: ASR, age‐ and sex‐standardised mortality rate; CI, confidence interval; CMR, crude mortality rate; PY, person years.

^a^ Standardised mortality rate not estimable because total deaths <20 and/or number of people in at least one age‐sex stratum <20.

In‐treatment mortality rates were significantly lower than post‐treatment mortality rates for counselling and residential rehabilitation. A pattern of lower in‐treatment CMR point estimates was also observed for IDAT and outpatient consultation, but as confidence intervals overlapped statistical significance is uncertain. In‐treatment CMR point estimates did not differ substantially from post‐treatment CMRs for support and case management and for withdrawal management, and confidence intervals overlapped substantially.

In‐treatment mortality rates were significantly higher for counselling, outpatient consultation, support and case management and withdrawal management than for residential rehabilitation. IDAT could not be included in this comparison due to limited in‐treatment person‐time and deaths. In‐treatment ASRs could be computed only for counselling, preventing us from adjusting for potential age and sex differences in our comparison across treatment modalities.

We found significantly higher post treatment mortality rates among people who previously received IDAT, outpatient consultation and withdrawal management, relative to all other treatment modalities. Post‐treatment mortality was significantly higher for IDAT than outpatient consultation, which in turn was associated with higher post‐treatment mortality than withdrawal management. After age‐ and sex‐standardisation, post‐treatment mortality rates remained elevated for outpatient consultation relative to other modalities, while post‐treatment ASRs for withdrawal management were significantly higher than those for counselling and residential rehabilitation only. IDAT could not be included in the age‐ and sex‐standardised comparisons owing to small cell counts.

Our sensitivity analyses among people with alcohol problems yielded results that were consistent with the main analysis (see Table S5a), although in some cases, a previously statistically significant difference was no longer significant. This is not surprising given the sensitivity analyses included fewer people and/or less person time, and we do not consider this a meaningful change to the study results.

### 3.2 Amphetamine‐type stimulants and cannabinoids

Among people with problems with amphetamine‐type stimulants or cannabinoids, mortality rates during and after treatment were computed for six treatment modalities (Table 3), with IDAT, OAT and rehabilitation excluded. For both these PDoCs, there was no evidence to suggest a significant difference between in‐treatment and post‐treatment CMRs, with estimates not differing markedly, and confidence intervals overlapping substantially for all treatment modalities.

**TABLE 3:** Crude mortality rates by treatment modality for people accessing treatment with amphetamine‐type stimulants or cannabinoids as the principal drug of concern in New South Wales, 2012–2018.

	Amphetamine‐type stimulants			Cannabinoids		
	Person years	Deaths	CMR per 100 PY [95% CI]	Person years	Deaths	CMR per 100 PY [95% CI]
Assessment only						
In treatment	N/A	N/A	N/A	N/A	N/A	N/A
Post treatment	6849	26	0.38 [0.25–0.56]	4895	19	0.39 [0.23–0.61]
Counselling						
In treatment	3733	8	0.21 [0.09–0.42]	^a^	<5	0.12 [0.03–0.31]
Post treatment	16,090	75	0.47 [0.37–0.58]	20,424	60	0.29 [0.22–0.38]
Outpatient consultation						
In treatment	^a^	<5	0.50 [0.01–2.77]	^a^	<5	0.62 [0.02–3.48]
Post treatment	2205	14	0.64 [0.35–1.07]	2143	11	0.51 [0.26–0.92]
Residential rehabilitation						
In treatment	1054	0	0.00 [0.00–0.35]	348	0	0.00 [0.00–1.06]
Post treatment	6476	17	0.26 [0.15–0.42]	2586	7	0.27 [0.11–0.56]
Support and case management						
In treatment	^a^	<5	0.26 [0.07–0.67]	^a^	<5	0.07 [0.00–0.40]
Post treatment	7855	26	0.33 [0.22–0.48]	8847	20	0.23 [0.14–0.35]
Withdrawal management						
In treatment	89	0	0.00 [0.00–1.28]	^a^	<5	0.36 [0.01–1.99]
Post treatment	6074	32	0.53 [0.36–0.74]	5981	17	0.28 [0.17–0.46]

Abbreviations: CI, confidence interval; CMR, crude mortality rate; PY, person years.

^a^ Cell value suppressed so that the number of deaths cannot be determined, where small (<5) death counts pose a risk of re‐identification.

During‐treatment CMRs did not differ markedly across different treatment modalities among people with problems with amphetamine‐type stimulants or cannabinoids, and confidence intervals overlapped substantially. Small cell sizes meant we could not adjust for potential age and sex difference in this comparison (see Table S6).

For both these PDoCs, inspection of post‐treatment CMRs and the associated confidence intervals did not suggest marked differences across different treatment modalities. We were able to compute post‐treatment ASRs only for counselling and support and case management, as well as withdrawal management for amphetamine‐type stimulants; there was no evidence to suggest a significant difference in post‐treatment ASRs across these modalities.

All three sensitivity analyses among people with problems with amphetamine‐type stimulants or cannabinoids (Table S5b, c, respectively) yielded results that were consistent with the main analysis.

### 3.3 Opioids

Among people with opioid problems, mortality rates during and after treatment were computed for seven treatment modalities (Table 4), with IDAT and rehabilitation excluded. Comparisons of in‐treatment and post‐treatment mortality rates indicate that, for counselling and OAT, mortality during treatment was significantly lower than after treatment. A similar pattern was indicated for outpatient consultation and residential rehabilitation, although confidence intervals around these CMRs overlapped. In‐treatment CMRs did not differ markedly from post‐treatment CMRs for support and case management and for withdrawal management, and confidence intervals overlapped substantially.

**TABLE 4:** Crude mortality rates and age‐ and sex‐standardised mortality rates by treatment modality for people accessing treatment with opioids as the principal drug of concern in New South Wales, 2012–2018.

	Person years	Deaths	CMR per 100 PY [95% CI]	ASR per 100 PY [95%CI]
Assessment only				
In treatment	N/A	N/A	N/A	N/A
Post treatment	3460	25	0.72 [0.47–1.07]	1.00 [0.49–1.52]
Counselling				
In treatment	1862	8	0.43 [0.19–0.85]	^b^
Post treatment	7564	81	1.07 [0.85–1.33]	1.22 [0.86–1.58]
Opioid agonist treatment				
In treatment	20,460	172	0.84 [0.72–0.98]	0.85 [0.68–1.02]
Post treatment	22,483	273	1.21 [1.07–1.37]	1.36 [1.14–1.58]
Outpatient consultation				
In treatment	^a^	<5	1.09 [0.23–3.19]	^b^
Post treatment	1871	30	1.60 [1.08–2.29]	2.13 [1.28–2.98]
Residential rehabilitation				
In treatment	^a^	<5	0.19 [0.00–1.03]	^b^
Post treatment	2770	22	0.79 [0.50–1.20]	1.67 [0.00–3.35]
Support and case management				
In treatment	1160	14	1.21 [0.66–2.03]	^b^
Post treatment	4028	29	0.72 [0.48–1.03]	0.82 [0.35–1.30]
Withdrawal management				
In treatment	464	5	1.08 [0.35–2.51]	^b^
Post treatment	5901	71	1.20 [0.94–1.52]	1.36 [0.90–1.82]

Abbreviations: ASR, age‐ and sex‐standardised mortality rate; CI, confidence interval; CMR, crude mortality rate; PY, person years.

^a^ Cell value suppressed so that the number of deaths cannot be determined, where small (<5) death counts pose a risk of re‐identification.

^b^ Standardised mortality rate not estimable because total deaths <20 and/or number of people in at least one age‐sex stratum <20.

Among people with opioid problems, a pattern of higher in‐treatment CMRs was seen for all treatment modalities relative to counselling and residential rehabilitation, but confidence intervals around all in‐treatment CMRs overlapped. Small cell counts meant an in‐treatment age‐ and sex‐standardised mortality rate could be computed only for OAT.

Post‐treatment CMRs for assessment only and support and case management were significantly lower than those for OAT and outpatient consultation. After age‐ and sex‐standardisation, this pattern remained for outpatient consultation only, however, confidence intervals overlapped.

Our sensitivity analyses among people with opioid problems yielded results that were mostly consistent with the main analysis (Table S5d), although some differences observed in the main analysis were not always accompanied by non‐overlapping confidence intervals in sensitivity analyses. The analysis restricted to treatment received between July 2015 and December 2018, however, did yield a contrasting result with no difference found between the in‐treatment and post‐treatment CMR for OAT, both in terms of similar CMR point estimates and overlapping confidence intervals.

## 4 DISCUSSION

To our knowledge, this is the first comprehensive study examining mortality among people accessing different specialist alcohol and other drug treatment modalities for several drugs of concern. We found significantly higher mortality rates among people accessing treatment for problems with alcohol or opioids, relative those with problems with amphetamine‐type stimulants or cannabinoids. This pattern persisted after age‐ and sex‐standardisation. We also found differences in mortality rates according to treatment status and treatment modality among people with alcohol or opioid problems. We found no such differences among people with problems with amphetamine‐type stimulants or cannabinoids, probably due in part to the low overall mortality rate in these groups and limited statistical power to detect differences, but potentially also due to the limited effectiveness of existing treatments for stimulant use disorders [26]. These findings highlight the importance of examining mortality rates separately by PDoC when examining variation in mortality rates. They also suggest there is scope to reduce mortality among people accessing treatment with alcohol or opioid problems. Larger scale studies are needed to determine whether this is also the case for people accessing treatment with amphetamine‐type stimulants or cannabinoids as their drug of concern.

Our comparisons of mortality during and after treatment point to service delivery and policy changes that might contribute to reduced mortality risk among people accessing treatment for problems with alcohol or opioids. Consistent evidence that engagement in OAT is associated with reduced mortality has led to calls for expanded access to OAT, strategies to maximise retention, and/or greater post‐treatment follow‐up [5, 6, 27]. Similar strategies should be considered for treatment modalities where mortality rates were reduced during treatment, relative to after treatment: counselling, residential rehabilitation, IDAT and outpatient consultation for people with problematic use of alcohol; and counselling, OAT, outpatient consultation and residential rehabilitation for people with opioid problems. It is important to note, however, that these are associations and we have not formally investigated causality. Establishing causality is extremely challenging in this setting where randomised studies are impractical and unethical [5, 6], and non‐randomised studies, including the current study, are subject to confounding by factors that are related to both the decision to participate in treatment (and the treatment modality chosen) and the risk of mortality [8]. Likely confounding factors include severity of the person's substance dependence, the extent to which they have problems with other drugs, their physical and mental health, their financial, housing and employment circumstances, and the strength of their support networks. It should be the goal of future research to obtain good quality data on likely confounding factors and to conduct robust causal analyses. While there is a dearth of evidence from causal studies, it is important to carefully evaluate the impact of any implemented policy reform or service changes so that they can be reversed, modified or extended based on the findings of the evaluation.

In implementing strategies to optimise retention and/or post‐treatment follow‐up, there could be merit in prioritising outpatient consultation for people with opioid problems given they had higher post‐treatment mortality relative to people accessing other modalities even after adjusting for age and sex. The same applies to outpatient consultation and withdrawal management for people with alcohol problems. Among people accessing treatment for alcohol problems, there was also variation between modalities in during‐treatment mortality, suggesting there may be value in adjunct interventions for people accessing certain treatment modalities for alcohol problems (counselling, outpatient consultation, support and case management, and withdrawal management). However, as we could not adjust for age and sex in this comparison, this result should be interpreted with caution. Although these between‐modality differences in mortality likely reflect variation in the underlying mortality risk among people accessing these different modalities (the distribution of risk factors in our cohort varied by treatment modality), this variation does suggest the mortality risk in this population is to some extent modifiable. Research building an understanding of how these and other risk factors relate to mortality risk in people accessing different treatment modalities may inform the design of interventions. Similarly, appropriately powered research investigating the causes of death occurring in these groups with elevated mortality risk would provide insight into the common pathways to mortality. Overdose is the most common cause of death among people with opioid dependence [3], while liver cirrhosis, mental health disorders and injuries account for a large proportion of the excess mortality among people with alcohol use disorders [28]. Whether this holds across all treatment modalities and in‐treatment and post‐treatment periods remains to be elucidated.

### 4.1 Study limitations

Our identification of in‐treatment and post‐treatment periods is imperfect. Although the MDS DATS covers NSW Ministry of Health funded specialist alcohol and other treatment services, treatment for alcohol and other drug problems is also provided in other settings including private treatment agencies, inpatient and outpatient services without a specialisation in alcohol and other drug treatment and services provided in correctional settings. Additionally, while we had data on non‐specialist outpatient services through the NAP data collection, we did not include these episodes in the analysis because the PDoC could not be identified. These data coverage issues mean that, during person‐time we classified as in‐treatment and/or post‐treatment, our cohort may have received services we did not identify. OAT is the modality most impacted by these data coverage issues, with only one‐third of OAT episodes in NSW managed by services receiving NSW Ministry of Health funding [29]. Additionally, OAT episodes can be long in duration and were therefore more likely to still be open at the time of data extraction. The lack of data on open treatment episodes likely led to a greater risk of misclassification later in the study period, with more person‐time incorrectly classified as post‐treatment instead of in‐treatment. This may account for the inconsistent finding between the main analysis and the 2015–2018 sensitivity analyses in relation to OAT. As the 2015–2018 analysis did not yield altered conclusions for other treatment modalities or other PDoCs, the inconsistent finding is unlikely due to improved linkage accuracy in recent years. Given the high risk of misclassified person‐time arising from incomplete identification of OAT episodes, the results relating to mortality during and after treatment for people with opioid problems should be interpreted with caution.

As we used the non‐overlap of confidence intervals as an indicator of statistical significance, uncertainty remains as to whether those estimates with overlapping confidence intervals were statistically significantly different from each other. In these cases, we based our interpretation on inspection of the CMR/ASR estimates and the width of the confidence intervals. While this method can be subjective, we considered it reasonable given the growing acceptance in the scientific community that statistical interpretation and reporting would be improved by focusing on estimation (the size of effects and the uncertainty surrounding those estimates) instead of statistical testing [25]. It is also important to note that mortality rates are one measure of the harms experienced by people with alcohol or other drug problems. Future research measuring the relationship between treatment and alcohol and other drug‐related harm, and how the risk of harm varies across treatment modalities, might consider a more comprehensive range of outcomes including substance use, physical and mental health, and quality of life, as well as social outcomes.

### 4.2 Conclusions

Mortality rates differed according to treatment status and treatment modality among people with alcohol or opioid problems, indicating there is scope to reduce mortality among people accessing treatment for these drugs of concern. Larger‐scale studies are needed to determine whether this is also the case for people accessing treatment for problems with amphetamine‐type stimulants or cannabinoids. Research to inform the design of appropriate responses to the elevated mortality among people accessing drug and alcohol treatment should account for the variation in mortality by PDoC and treatment modality.

## AUTHOR CONTRIBUTIONS

Each author certifies that their contribution to this work meets the standards of the International Committee of Medical Journal Editors.

## CONFLICT OF INTEREST STATEMENT

Louisa Degenhardt and Michael Farrell have received investigator‐initiated untied educational grants from Reckitt Benckiser/Indivior, Mundipharma and Seqirus Pty Ltd. Michael Farrell is also on the Board of Directors for a specialist alcohol and drug treatment service in NSW. The Centre for Big Data Research in Health at UNSW Sydney received funding from AbbVie Australia in 2020. These grants were unrelated to the current study, and none of these companies had any knowledge or involvement in this study. All other authors declare no competing interests.

## Supporting information


**Data S1:** Supporting information

## References

[1] Roerecke M , Rehm J . Alcohol use disorders and mortality: a systematic review and meta‐analysis. Addiction. 2013;108:1562–78. 23627868 10.1111/add.12231

[2] Stockings E , Tran LT , Santo T Jr , Peacock A , Larney S , Santomauro D , et al. Mortality among people with regular or problematic use of amphetamines: a systematic review and meta‐analysis. Addiction. 2019;114:1738–50. 31180607 10.1111/add.14706 PMC6732053

[3] Degenhardt L , Bucello C , Mathers B , Briegleb C , Ali H , Hickman M , et al. Mortality among regular or dependent users of heroin and other opioids: a systematic review and meta‐analysis of cohort studies. Addiction. 2011;106:32–51. 21054613 10.1111/j.1360-0443.2010.03140.x

[4] Calabria B , Degenhardt L , Hall W , Lynskey M . Does cannabis use increase the risk of death? Systematic review of epidemiological evidence on adverse effects of cannabis use. Drug Alcohol Rev. 2010;29:318–30. 20565525 10.1111/j.1465-3362.2009.00149.x

[5] Santo T Jr , Clark B , Hickman M , Grebely J , Campbell G , Sordo L , et al. Association of opioid agonist treatment with all‐cause mortality and specific causes of death among people with opioid dependence: a systematic review and meta‐analysis. JAMA Psychiatry. 2021;78:979–93. 34076676 10.1001/jamapsychiatry.2021.0976 PMC8173472

[6] Davoli M , Bargagli A , Perucci C , Schifano P , Belleudi V , Hickman M , et al. Risk of fatal overdose during and after specialist drug treatment: the VEdeTTE study, a national multi‐site prospective cohort study. Addiction. 2007;102:1954–9. 18031430 10.1111/j.1360-0443.2007.02025.x

[7] Evans E , Li L , Min J , Huang D , Urada D , Liu L , et al. Mortality among individuals accessing pharmacological treatment for opioid dependence in California, 2006‐10. Addiction. 2015;110:996–1005. 25644938 10.1111/add.12863 PMC4452110

[8] Pierce M , Bird SM , Hickman M , Marsden J , Dunn G , Jones A , et al. Impact of treatment for opioid dependence on fatal drug‐related poisoning: a national cohort study in England. Addiction. 2016;111:298–308. 26452239 10.1111/add.13193 PMC4950033

[9] Arendt M , Munk‐Jørgensen P , Sher L , Jensen SO . Mortality following treatment for cannabis use disorders: predictors and causes. J Subst Abus Treat. 2013;44:400–6. 10.1016/j.jsat.2012.09.007 23122774

[10] Lloyd B , Zahnow R , Barratt MJ , Best D , Lubman DI , Ferris J . Exploring mortality among drug treatment clients: the relationship between treatment type and mortality. J Subst Abus Treat. 2017;82:22–8. 10.1016/j.jsat.2017.09.001 29021111

[11] Degenhardt L , Randall D , Hall W , Law M , Butler T , Burns L . Mortality among clients of a state‐wide opioid pharmacotherapy program over 20 years: risk factors and lives saved. Drug Alcohol Depend. 2009;105:9–15. 19608355 10.1016/j.drugalcdep.2009.05.021

[12] Tisdale C , De Andrade D , Leung J , Chiu V , Hides L . Utilising data linkage to describe and explore mortality among a retrospective cohort of individuals admitted to residential substance abuse treatment. Drug Alcohol Rev. 2021;40:1202–6. 34590362 10.1111/dar.13279

[13] Arendt M , Munk‐Jørgensen P , Sher L , Jensen SO . Mortality among individuals with cannabis, cocaine, amphetamine, MDMA, and opioid use disorders: a nationwide follow‐up study of Danish substance users in treatment. Drug Alcohol Depend. 2011;114:134–9. 20971585 10.1016/j.drugalcdep.2010.09.013

[14] Maughan BC , Becker EA . Drug‐related mortality after discharge from treatment: a record‐linkage study of substance abuse clients in Texas, 2006‐2012. Drug Alcohol Depend. 2019;204:107473. 31520924 10.1016/j.drugalcdep.2019.05.011

[15] Australian Bureau of Statistics . Australian Standard Classification of Drugs of Concern [Internet]. Canberra: ABS; 2011. [cited 23 October 2021]. Available from: https://www.abs.gov.au/AUSSTATS/abs@.nsf/allprimarymainfeatures/BE7235FDCA6B6362CA25761C0019CD78?opendocument

[16] Australian Bureau of Statistics . Australian Standard Classification of Drugs of Concern [Internet]. Canberra: ABS; 2011 [cited 23 October 2021]. Available from: https://www.abs.gov.au/statistics/classifications/australian-standard-classification-drugs-concern/latest-release.

[17] National Centre for Classification in Health . The International Statistical Classification of Diseases and Related Health Problems, Tenth Revision, Australian Modification (ICD‐10‐AM). 5th ed. Sydney: Faculty of Health Sciences, The University of Sydney; 2008.

[18] National Centre for Classification in Health . The Australian classification of health interventions (ACHI). Seventh ed. Sydney: Faculty of Health Sciences, The University of Sydney; 2010.

[19] Independent Hospital Pricing Authority . Australian refined diagnosis related groups version 10.0 2019. [cited 22 October 2021]. Available from: https://www.ihpa.gov.au/what-we-do/admitted-acute-care/ar-drg-version-10

[20] Independent Hospital Pricing Authority . Tier 2 non‐admitted services 2018–19 2019. [cited 22 October 2021]. Available from: https://www.ihpa.gov.au/publications/tier-2-non-admitted-services-2018-19

[21] Health System Information & Performance Reporting . Data dictionary and collection requirements for the NSW minimum data set for drug and alcohol treatment services. Sydney: NSW Ministry of Health; 2015.

[22] Washington State Department of Health . Guide for using confidence intervals for public health assessment. 2012 [cited 23 March 2023]. Available from: https://doh.wa.gov/sites/default/files/legacy/Documents/1500/ConfIntGuide.pdf.

[23] Australian Bureau of Statistics . Deaths, year of occurrence, age at death, age‐specific death rates, sex, states, Territories and Australia [Data Explorer] 2020 [cited 10 January 2022]. Available from: https://explore.data.abs.gov.au/vis?fs[0]=ABS%20Topics%2C1%7CPEOPLE%23PEOPLE%23%7CPopulation%23POPULATION%23&fs[1]=Measure%2C0%7CDeaths%234%23&pg=0&fc=Measure&df[ds]=ABS_ABS_TOPICS&df[id]=DEATHS_AGESPECIFIC_OCCURENCEYEAR&df[ag]=ABS&df[vs]=1.0.0&pd=2009%2C&dq=4.3..AUS.A&ly[cl]=TIME_PERIOD&ly[rw]=AGE

[24] Berry J , Harrison J . A guide to statistical methods for injury surveillance. Injury technical paper series No 5. (AIHW cat. no. INJCAT 72). Adelaide: AIHW; 2006.

[25] Greenland S , Senn S , Rothman K , Carlin J , Poole C , Goodman S , et al. Statistical tests, P values, confidence intervals, and power: a guide to misinterpretations. Eur J Epidemiol. 2016;31:331–50. 27209009 10.1007/s10654-016-0149-3 PMC4877414

[26] Ronsley C , Nolan S , Knight R , Hayashi K , Klimas J , Walley A , et al. Treatment of stimulant use disorder: a systematic review of reviews. PLoS One. 2020;15:e0234809. 32555667 10.1371/journal.pone.0234809 PMC7302911

[27] Ma J , Bao Y‐P , Wang R‐J , Su M‐F , Liu M‐X , Li J‐Q , et al. Effects of medication‐assisted treatment on mortality among opioids users: a systematic review and meta‐analysis. Mol Psychiatry. 2019;24:1868–83. 29934549 10.1038/s41380-018-0094-5

[28] Roerecke M , Rehm J . Cause‐specific mortality risk in alcohol use disorder treatment patients: a systematic review and meta‐analysis. Int J Epidemiol. 2014;43:906–19. 24513684 10.1093/ije/dyu018

[29] Australian Institute of Health and Welfare. National Opioid Pharmacotherapy Statistics Annual Data collection . Cat. no. PHE 266 Canberra: AIHW; 2021 [cited 2 December 2021]. Available from: https://www.aihw.gov.au/reports/alcohol‐other‐drug‐treatment‐services/national‐opioidpharmacotherapy‐statistics

