# Supplementary material for: Mortality during and after specialist alcohol and other drug treatment: Variation in rates according to principal drug of concern and treatment modality
Source: Drug Alcohol Rev. 2023 Apr 25;42(6):1461–71. doi: 10.1111/dar.13669 (PMC10946946; doi:10.1111/dar.13669)
Supplement: Supplementary file 1 — Data S1: Supporting information [file DAR-42-1461-s001.docx]

**Supporting Information**

[Table S1. Mapping of original service type categories in MDS DATS data to treatment modalities considered in this study 2](#_Toc132054436)

[Table S2. Codes used to identify episodes of withdrawal management for alcohol use disorder from hospital admission records; admissions were indicated when at least one of the relevant ACHI or AR-DRG codes was recorded as well as a relevant ICD-10-AM code 3](#_Toc132054437)

[Table S3. Mapping of original principal drug of concern codes in Minimum Data Set for Drug and Alcohol Treatment Services to the four principal drug of concern categories used in the current study 4](#_Toc132054438)

[Figure S1. Flowchart of cohort inclusions and exclusions 5](#_Toc132054439)

[Table S4a. Cohort characteristics – Alcohol 6](#_Toc132054440)

[Table S4b. Cohort characteristics – Amphetamine-type stimulants 7](#_Toc132054441)

[Table S4c. Cohort characteristics – Cannabinoids 8](#_Toc132054442)

[Table S4d. Cohort characteristics – Opioids 10](#_Toc132054443)

[Table S5a. Results of sensitivity analyses – Alcohol 12](#_Toc132054444)

[Table S5b. Results of sensitivity analyses – Amphetamine-type stimulants 13](#_Toc132054445)

[Table S5c. Results of sensitivity analyses – Cannabinoids 14](#_Toc132054446)

[Table S5d. Results of sensitivity analyses – Opioids 15](#_Toc132054447)

[Table S6. Age- and sex- standardised mortality rates for amphetamine-type stimulants and cannabinoids, per 100 PY [95% CI] 16](#_Toc132054448)

## Table S1. Mapping of original service type categories in MDS DATS data to treatment modalities considered in this study

| **Current study treatment modalities** | **Main service provided, as entered in MDS DATS**^a^ | **Delivery setting** |
| --- | --- | --- |
| **Assessment only** | Assessment only | All |
| **Counselling** | Counselling | All |
| **Involuntary Drug and Alcohol Treatment** | Involuntary Drug and Alcohol Treatment | All |
| **Opioid agonist treatment** | Maintenance pharmacotherapy (opioid) | All |
| **Outpatient consultation** | Consultation activities  Outpatient consultation (pre-2015 code) | All except inpatient  All |
| **Rehabilitation** | Rehabilitation activities  Day rehabilitation activities (pre-2015 code) | All except inpatient  All |
| **Residential rehabilitation** | Rehabilitation activities  Residential rehabilitation activities (pre-2015 code) | Inpatient  All |
| **Support and case management** | Support and case management only | All |
| **Withdrawal management** | Withdrawal management (detoxification)  Inpatient/residential withdrawal management (pre-2015 code)  Outpatient withdrawal management (pre-2015) | All  All  All |
| **Excluded from this study due to inconsistent recording between and within agencies^b^** | Information and education only | All |
|  | Consultation activities  Inpatient consultation (pre-2015 code) | Inpatient  All |
|  | Maintenance pharmacotherapy (non-opioid) | All |
|  | Other  Unknown codes | All  All |

MDS DATS, Minimum Data Set for Drug and Alcohol Treatment Services; NSW, New South Wales.

^a^ Descriptions of each of these services can be found in the NSW MDS Data Dictionary (Health System Information and Performance Reporting, Data Dictionary and Collection Requirements for the NSW Minimum Data Set for Drug and Alcohol Treatment Services. NSW Ministry of Health: Sydney 2015. Available at: https://www1.health.nsw.gov.au/pds/Pages/doc.aspx?dn=PD2015_014). ^b^ Our analyses excluded any in-treatment and post-treatment person-time relating to these excluded treatment modalities.

## Table S2. Codes used to identify episodes of withdrawal management for alcohol use disorder from hospital admission records; admissions were indicated when at least one of the relevant ACHI or AR-DRG codes was recorded as well as a relevant ICD-10-AM code

| **ACHI code in primary procedure field** | **AR-DRG** | |
| --- | --- | --- |
| **92002-00** Alcohol rehabilitation  **92003-00** Alcohol detoxification  **92004-00** Alcohol rehabilitation and detoxification  **92008-00** Combined alcohol and drug rehabilitation  **92009-00** Combined alcohol and drug detoxification  **92010-00** Combined alcohol and drug rehabilitation | **OR** | **V60 (A and B)** Alcohol intoxication and withdrawal  **V62 (A and B)** Alcohol dependence and withdrawal |
| **AND ICD-10-AM code in principal diagnosis field** | | |
| **F10.1** Mental and behavioural disorders due to use of alcohol, harmful use  **F10.2** Mental and behavioural disorders due to use of alcohol, dependence syndrome  **F10.3** Mental and behavioural disorders due to use of alcohol, withdrawal state  **F10.4** Mental and behavioural disorders due to use of alcohol, withdrawal state with delirium  **Z50.2** Alcohol rehabilitation  **Z72.1** Alcohol use | | |

ACHI, Australian Classification of Health Interventions; AR-DRG, Australian-Refined Diagnostic Related Groups; ICD-10-AM, International Statistical Classification of Diseases and Related Health Problems, Tenth Revision, Australian Modification

## Table S3. Mapping of original principal drug of concern codes in Minimum Data Set for Drug and Alcohol Treatment Services to the four principal drug of concern categories used in the current study

| **Principal drug of concern categories** | **Australian Standard Classification of Drugs of Concern code** | **Australian Standard Classification of Drugs of Concern description** |
| --- | --- | --- |
| **Alcohol** | 2100-2199 | Sedatives and hypnotics – alcohols |
| **Amphetamine-type stimulants** | 0006 | Psychostimulants not further defined |
|  | 3000 | Stimulants and hallucinogens not further defined |
|  | 3100-3199 | Stimulants and hallucinogens – amphetamines |
|  | 3300-3399 | Stimulants and hallucinogens – ephedra alkaloids |
|  | 3400-3499 | Stimulants and hallucinogens – phenethylamines |
|  | 3903 | Cocaine |
|  | 3905 | Methylphenidate |
|  | 3999 | Other stimulants and hallucinogens not elsewhere classified |
| **Cannabinoids** | 3201 (code retired in 2015) | Cannabinoids |
|  | 7100-7199 | Cannabinoids and related drugs |
| **Opioids** | 0005 | Pharmaceutical opioids not further defined |
|  | 1000 | Analgesics not further defined |
|  | 1100-1199 | Organic opiate analgesics |
|  | 1200-1203, 1299 | Semisynthetic opioid analgesics |
|  | 1300-1399 | Synthetic opioid analgesics |

## Figure S1. Flowchart of cohort inclusions and exclusions


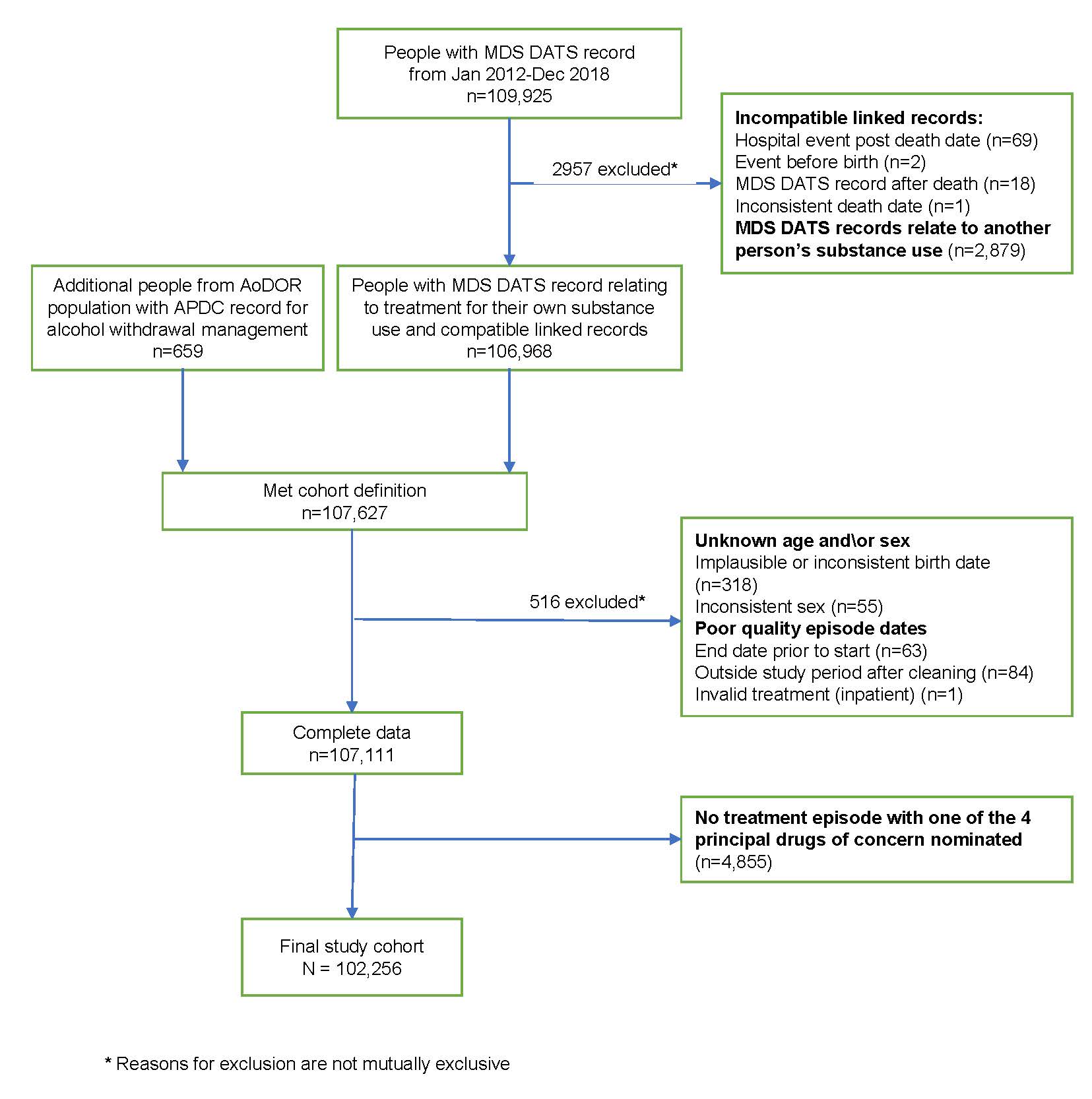


AoDOR, Alcohol and Other Drugs Outcomes Register; APDC, Admitted Patient Data Collection; MDS DATS, Minimum Data Set for Drug and Alcohol Treatment Services.

## Table S4a. Cohort characteristics – Alcohol

|  |  |  |  |  | **Accommodation immediately prior to episode** | | | |  | **Past 12-month hospital admissions** | | |  |
| --- | --- | --- | --- | --- | --- | --- | --- | --- | --- | --- | --- | --- | --- |
|  | **People** | **Episodes** | **Male (%)** | **Age at treatment entry in years (median [IQR])** | **Home rented or owned (%)** | **Health, justice or supervised facility (%)** | **No usual residence (%)** | **Short-term, other or not known (%)** | **Injecting drug use in last 12 months (%)** | **Mental health^a^ (%)** | **Substance use^b^ (%)** | **Self-harm^c^ (%)** | **Episode length in days (median [IQR])** |
| Assessment only | 8050 | 8731 | 69.1 | 41 [32-49] | 74 | 6 | 4 | 16 | 10 | 15 | 32 | 6 | 1 [1-1] |
| Counselling | 21,509 | 25,607 | 66.4 | 41 [32-50] | 81 | 1 | 1 | 16 | 3 | 15 | 34 | 6 | 58  [18-124] |
| IDAT | 193 | 225 | 53.4 | 47 [39-54] | 70 | 5 | 9 | 16 | 6 | 61 | 99 | 34 | 41  [28-73] |
| OAT | 144 | 145 | 63.9 | 45 [39-52] | 74 | ^d^ | ^d^ | 22 | 15 | 21 | 60 | 13 | 86  [9-426] |
| Outpatient consultation | 4386 | 4912 | 63.6 | 45 [36-54] | 78 | 4 | 2 | 16 | 6 | 22 | 61 | 12 | 5 [1-41] |
| Rehabilitation | 634 | 715 | 51.4 | 42 [34-49] | 84 | 5 | 2 | 8 | 6 | 26 | 49 | 10 | 52  [17-93] |
| Residential rehabilitation | 3892 | 4234 | 65.9 | 40 [32-47] | 70 | 15 | 7 | 8 | 11 | 19 | 46 | 8 | 57  [22-121] |
| Support and case management | 5632 | 6140 | 66.9 | 41 [32-50] | 78 | 3 | 5 | 15 | 5 | 21 | 43 | 9 | 41  [9-106] |
| Withdrawal management | 14,946 | 26,361 | 65.7 | 43 [35-52] | 34 | 1 | 3 | 63 | 9 | 23 | 77 | 9 | 7 [4-9] |

IDAT, Involuntary Drug and Alcohol Treatment; IQR, interquartile range; OAT, opioid agonist treatment.

^a^ Defined as presence of any of the following ICD-10-AM codes in any diagnosis field of APDC records in prior 12 months: F20, F22-F25, F28, F29, F30.2, F31.2, F31.5, F20.4, F31.3, F31.4, F31.5, F32, F33, F34.1, F41.2, F43.2. ^b^ Defined as presence of ICD-10-AM codes F10 to F19 in any diagnosis field. ^c^ Defined as presence of ICD-10-AM codes X60 to X84 in any diagnosis field; ^d^ Cell value suppressed because based on <5 individuals, posing a risk of re-identification.

## Table S4b. Cohort characteristics – Amphetamine-type stimulants

|  |  |  |  |  | **Accommodation immediately prior to episode** | | | |  | **Past 12-month hospital admissions** | | |  |
| --- | --- | --- | --- | --- | --- | --- | --- | --- | --- | --- | --- | --- | --- |
|  | **People** | **Episodes** | **Male (%)** | **Age at treatment entry in years (median [IQR])** | **Home rented or owned (%)** | **Health, justice or supervised facility (%)** | **No usual residence (%)** | **Short-term, other or not known (%)** | **Injecting drug use in last 12 months (%)** | **Mental health^a^ (%)** | **Substance use^b^ (%)** | **Self-harm^c^ (%)** | **Episode length in days (median [IQR])** |
| Assessment only | 7321 | 7908 | 69.1 | 31 [26-38] | 70 | 13 | 6 | 11 | 43 | 13 | 30 | 6 | 1 [1-1] |
| Counselling | 11,556 | 12,857 | 67.8 | 32 [26-39] | 80 | 3 | 3 | 14 | 36 | 15 | 31 | 7 | 64 [22-120] |
| IDAT | 36 | 37 | 33.3 | 33 [28-39] | 58 | ^d^ | 22 | ^d^ | 67 | 78 | 97 | 19 | 40 [17-81] |
| OAT | 536 | 546 | 67.4 | 37 [31-43] | 72 | 9 | 3 | 16 | 71 | 20 | 48 | 10 | 158 [41-443] |
| Outpatient consultation | 2061 | 2140 | 65.0 | 33 [27-40] | 77 | 4 | 5 | 13 | 46 | 18 | 51 | 9 | 2 [1-34] |
| Rehabilitation | 780 | 887 | 56.4 | 33 [28-39] | 79 | 5 | 4 | 12 | 42 | 15 | 39 | 8 | 46 [22-80] |
| Residential rehabilitation | 4302 | 4689 | 65.8 | 32 [26-38] | 71 | 13 | 9 | 8 | 49 | 15 | 40 | 7 | 42 [14-97] |
| Support and case management | 6009 | 6319 | 69.2 | 32 [25-39] | 79 | 3 | 6 | 12 | 43 | 15 | 35 | 7 | 59 [17-98] |
| Withdrawal management | 4695 | 5432 | 64.5 | 33 [27-39] | 70 | 6 | 10 | 14 | 50 | 19 | 63 | 9 | 6 [3-9] |

IDAT, Involuntary Drug and Alcohol Treatment; IQR, interquartile range; OAT, opioid agonist treatment.

^a^ Defined as presence of any of the following ICD-10-AM codes in any diagnosis field of APDC records in prior 12 months: F20, F22-F25, F28, F29, F30.2, F31.2, F31.5, F20.4, F31.3, F31.4, F31.5, F32, F33, F34.1, F41.2, F43.2. ^b^ Defined as presence of ICD-10-AM codes F10 to F19 in any diagnosis field. ^c^ Defined as presence of ICD-10-AM codes X60 to X84 in any diagnosis field. ^d^ Cell value suppressed because based on <5 individuals, posing a risk of re-identification.

## Table S4c. Cohort characteristics – Cannabinoids

|  |  |  |  |  | **Accommodation immediately prior to episode** | | | |  | **Past 12-month hospital admissions** | | |  |
| --- | --- | --- | --- | --- | --- | --- | --- | --- | --- | --- | --- | --- | --- |
|  | **People** | **Episodes** | **Male (%)** | **Age at treatment entry in years (median [IQR])** | **Home rented or owned (%)** | **Health, justice or supervised facility (%)** | **No usual residence (%)** | **Short-term, other or not known (%)** | **Injecting drug use in last 12 months (%)** | **Mental health^a^ (%)** | **Substance use^b^ (%)** | **Self-harm^c^ (%)** | **Episode length in days (median [IQR])** |
| Assessment only | 4204 | 4411 | 72.2 | 29 [23-37] | 80 | 3 | 4 | 13 | 14 | 13 | 26 | 5 | 1 [1-1] |
| Counselling | 11,728 | 12,640 | 69.0 | 27 [21-37] | 79 | 2 | 2 | 17 | 7 | 14 | 25 | 5 | 57  [19-112] |
| IDAT | <5 | ^d^ | ^d^ | ^d^ | ^d^ | ^d^ | ^d^ | ^d^ | ^d^ | ^d^ | ^d^ | ^d^ | ^d^ |
| OAT | 74 | 76 | 75.7 | 41 [36-46] | ^d^ | 3 | ^d^ | 18 | 42 | 18 | 53 | 8 | 133  [27-346] |
| Outpatient consultation | 1387 | 1468 | 58.1 | 30 [23-38] | 83 | 2 | 2 | 13 | 12 | 18 | 50 | 7 | 3 [1-29 |
| Rehabilitation | 290 | 338 | 55.2 | 32 [26-39] | 83 | 4 | 4 | 9 | 17 | 19 | 38 | 10 | 52  [23-94] |
| Residential rehabilitation | 1332 | 1397 | 67.0 | 27 [22-36] | 75 | 8 | 7 | 10 | 15 | 16 | 41 | 7 | 36  [10-89] |
| Support and case management | 5238 | 5332 | 69.4 | 26 [21-35] | 83 | 2 | 3 | 12 | 10 | 12 | 26 | 5 | 72  [23-106] |
| Withdrawal management | 3668 | 3987 | 64.7 | 30 [23-38] | 80 | 3 | 5 | 12 | 17 | 18 | 62 | 6 | 7 [4-12] |

IDAT, Involuntary Drug and Alcohol Treatment; IQR, interquartile range; OAT, opioid agonist treatment.

^a^ Defined as presence of any of the following ICD-10-AM codes in any diagnosis field of APDC records in prior 12 months: F20, F22-F25, F28, F29, F30.2, F31.2, F31.5, F20.4, F31.3, F31.4, F31.5, F32, F33, F34.1, F41.2, F43.2. ^b^ Defined as presence of ICD-10-AM codes F10 to F19 in any diagnosis field. ^c^ Defined as presence of ICD-10-AM codes X60 to X84 in any diagnosis field; ^d^ Cell value suppressed because based on <5 individuals, posing a risk of re-identification. ^d^ Cell value suppressed because based on <5 individuals, posing a risk of re-identification.

## Table S4d. Cohort characteristics – Opioids

|  |  |  |  |  | **Accommodation immediately prior to episode** | | | |  | **Past 12-month hospital admissions** | | |  |
| --- | --- | --- | --- | --- | --- | --- | --- | --- | --- | --- | --- | --- | --- |
|  | **People** | **Episodes** | **Male (%)** | **Age at treatment entry in years (median [IQR])** | **Home rented or owned (%)** | **Health, justice or supervised facility (%)** | **No usual residence (%)** | **Short-term, other or not known (%)** | **Injecting drug use in last 12 months (%)** | **Mental health^a^ (%)** | **Substance use^b^ (%)** | **Self-harm^c^ (%)** | **Episode length in days (median [IQR])** |
| Assessment only | 3548 | 3956 | 69.4 | 36 [30-43] | 75 | 7 | 5 | 13 | 63 | 10 | 32 | 5 | 1 [1-1] |
| Counselling | 4612 | 5218 | 62.8 | 37 [31-44] | 79 | 4 | 2 | 15 | 49 | 13 | 38 | 8 | 71  [20-152] |
| IDAT | 7 | 10 | 14.3 | 37 [30-42] | ^d^ | 0 | ^d^ | 0 | 86 | 71 | 100 | 29 | 18  [8-50] |
| OAT | 12,967 | 15,312 | 67.4 | 38 [32-46] | 72 | 4 | 3 | 21 | 53 | 10 | 28 | 5 | 242  [56-986] |
| Outpatient consultation | 1704 | 1815 | 57.7 | 40 [32-49] | 73 | 2 | 3 | 23 | 40 | 18 | 56 | 10 | 2 [1-39] |
| Rehabilitation | 164 | 177 | 60.4 | 36 [31-41] | 88 | 6 | 3 | 3 | 67 | 15 | 48 | 9 | 52  [23-97] |
| Residential rehabilitation | 1593 | 1753 | 67.7 | 36 [30-42] | 73 | 13 | 6 | 9 | 79 | 13 | 14 | 7 | 59  [21-114] |
| Support and case management | 2439 | 2515 | 63.1 | 36 [30-44] | 81 | 4 | 3 | 11 | 57 | 12 | 38 | 7 | 58  [16-147] |
| Withdrawal management | 3345 | 4069 | 64.2 | 37 [31-44] | 76 | 3 | 7 | 15 | 63 | 15 | 65 | 8 | 7 [4-10] |

IDAT, Involuntary Drug and Alcohol Treatment; IQR, interquartile range; OAT, opioid agonist treatment.

^a^ Defined as presence of any of the following ICD-10-AM codes in any diagnosis field of APDC records in prior 12 months: F20, F22-F25, F28, F29, F30.2, F31.2, F31.5, F20.4, F31.3, F31.4, F31.5, F32, F33, F34.1, F41.2, F43.2. ^b^ Defined as presence of ICD-10-AM codes F10 to F19 in any diagnosis field. ^c^ Defined as presence of ICD-10-AM codes X60 to X84 in any diagnosis field; ^d^ Cell value suppressed because based on <5 individuals, posing a risk of re-identification.

## Table S5a. Results of sensitivity analyses – Alcohol

|  | **CMR per 100 PY [95% CI]** | | | | | | |
| --- | --- | --- | --- | --- | --- | --- | --- |
|  | **Main analysis** | **2015-2018** | **Single services** | | **0-day rule** | | **7-day rule** |
| **Assessment only** |  |  |  | |  | |  |
| In treatment | N/A | N/A | | N/A | | N/A | N/A |
| Post treatment | 0.84 [0.66-1.05] | 1.06 [0.67-1.59] | 0.84 [0.66-1.06] | | N/A | | N/A |
| **Counselling** |  |  |  | |  | |  |
| In treatment | 0.44 [0.30-0.62] | 0.43 [0.24-0.72] | 0.39 [0.25-0.58] | | 0.44 [0.30-0.62] | | 0.52 [0.37-0.71] |
| Post treatment | 0.87 [0.78-0.97] | 1.17 [0.98-1.39] | 0.81 [0.72-0.91] | | 0.87 [0.78-0.97] | | 0.86 [0.77-0.95] |
| **IDAT** |  |  |  | |  | |  |
| In treatment | 0.00 [0.00-8.54] | 0.00 [0.00-9.08] | 0.00 [0.00-33.61] | | 0.00 [0.00-8.54] | | 2.32 [0.06-12.90] |
| Post treatment | 8.56 [3.44-17.64] | 9.38 [3.77-19.32] | 7.81 [0.95-28.21] | | 8.56 [3.44-17.64] | | 6.12 [1.99-14.28] |
| **Outpatient consultation** |  |  |  | |  | |  |
| In treatment | 0.87 [0.32-1.89] | 1.06 [0.29-2.71] | 1.17 [0.38-2.72] | | 0.58 [0.16-1.48] | | 1.44 [0.69-2.65] |
| Post treatment | 2.16 [1.82-2.56] | 2.67 [2.01-3.47] | 2.20 [1.80-2.66] | | 2.18 [1.83-2.58] | | 2.05 [1.72-2.44] |
| **Residential rehabilitation** |  |  |  | |  | |  |
| In treatment | 0.00 [0.00-0.30] | 0.00 [0.00-0.70] | 0.00 [0.00-0.45] | | 0.00 [0.00-0.30] | | 0.08 [0.00-0.45] |
| Post treatment | 0.57 [0.41-0.76] | 0.61 [0.31-1.09] | 0.56 [0.38-0.80] | | 0.57 [0.41-0.76] | | 0.55 [0.40-0.75] |
| **Support and case management** | |  |  | |  | |  |
| In treatment | 0.74 [0.37-1.32] | 0.74 [0.27-1.61] | 0.97 [0.47-1.78] | | 0.74 [0.37-1.32] | | 1.00 [0.56-1.66] |
| Post treatment | 0.96 [0.77-1.18] | 1.45 [1.01-2.00] | 0.84 [0.64-1.07] | | 0.96 [0.77-1.18] | | 0.87 [0.69-1.09] |
| **Withdrawal management** |  |  |  | |  | |  |
| In treatment | 0.89 [0.42-1.63] | 0.39 [0.05-1.39] | 0.93 [0.37-1.91] | | 0.71 [0.31-1.40] | | 1.68 [1.01-2.63] |
| Post treatment | 1.37 [1.24-1.52] | 2.41 [2.08-2.78] | 1.34 [1.19-1.51] | | 1.40 [1.26-1.55] | | 1.32 [1.18-1.46] |

CI, confidence interval; CMR, crude mortality rate; IDAT, Involuntary Drug and Alcohol Treatment; N/A, not applicable; PY, person years.

## Table S5b. Results of sensitivity analyses – Amphetamine-type stimulants

|  | **CMR per 100 PY [95% CI]** | | | | |
| --- | --- | --- | --- | --- | --- |
|  | **Main analysis** | **2015-2018** | **Single services** | **0-day rule** | **7-day rule** |
| **Assessment only** |  |  |  |  |  |
| In treatment | N/A | N/A | N/A | N/A | N/A |
| Post treatment | 0.38 [0.25-0.56] | 0.53 [0.30-0.85] | 0.38 [0.25-0.56] | N/A | N/A |
| **Counselling** |  |  |  |  |  |
| In treatment | 0.21 [0.09-0.42] | 0.18 [0.05-0.45] | 0.24 [0.10-0.49] | 0.21 [0.09-0.42] | 0.32 [0.17-0.56] |
| Post treatment | 0.47 [0.36-0.58] | 0.57 [0.41-0.78] | 0.44 [0.34-0.56] | 0.47 [0.37-0.58] | 0.45 [0.35-0.56] |
| **Outpatient consultation** |  |  |  |  |  |
| In treatment | 0.50 [0.01-2.77] | 0.68 [0.02-3.79] | 0.78 [0.02-4.37] | 0.50 [0.01-2.77] | 0.50 [0.01-2.77] |
| Post treatment | 0.64 [0.35-1.07] | 0.37 [0.10-0.95] | 0.74 [0.37-1.33] | 0.64 [0.35-1.07] | 0.64 [0.35-1.07] |
| **Residential rehabilitation** |  |  |  |  |  |
| In treatment | 0.00 [0.00-0.35] | 0.00 [0.00-0.59] | 0.00 [0.00-0.51] | 0.00 [0.00-0.35] | 0.00 [0.00-0.35] |
| Post treatment | 0.26 [0.15-0.42] | 0.31 [0.13-0.61] | 0.23 [0.12-0.41] | 0.26 [0.15-0.42] | 0.23 [0.13-0.38] |
| **Support and case management** | |  |  |  |  |
| In treatment | 0.26 [0.07-0.67] | 0.30 [0.06-0.88] | 0.26 [0.05-0.75] | 0.26 [0.07-0.67] | 0.33 [0.11-0.76] |
| Post treatment | 0.33 [0.22-0.48] | 0.44 [0.25-0.72] | 0.29 [0.17-0.45] | 0.33 [0.22-0.48] | 0.32 [0.21-0.47] |
| **Withdrawal management** |  |  |  |  |  |
| In treatment | 0.00 [0.00-1.28] | 0.00 [0.00-3.09] | 0.00 [0.00-1.79] | 0.00 [0.00-1.28] | 0.00 [0.00-1.28] |
| Post treatment | 0.53 [0.36-0.74] | 0.90 [0.58-1.34] | 0.48 [0.29-0.76] | 0.53 [0.36-0.74] | 0.51 [0.35-0.72] |

CI, confidence interval; CMR, crude mortality rate; N/A, not applicable; PY, person years.

## Table S5c. Results of sensitivity analyses – Cannabinoids

|  | **CMR per 100 PY [95% CI]** | | | | |
| --- | --- | --- | --- | --- | --- |
|  | **Main analysis** | **2015-2018** | **Single services** | **0-day rule** | **7-day rule** |
| **Assessment only** |  |  |  |  |  |
| In treatment | N/A | N/A | N/A | N/A | N/A |
| Post treatment | 0.39 [0.23-0.61] | 0.53 [0.21-1.10] | 0.39 [0.23-0.61] | N/A | N/A |
| **Counselling** |  |  |  |  |  |
| In treatment | 0.12 [0.03-0.31] | 0.06 [0.00-0.33] | 0.14 [0.04-0.37] | 0.12 [0.03-0.31] | 0.18 [0.07-0.39] |
| Post treatment | 0.29 [0.22-0.38] | 0.33 [0.21-0.50] | 0.25 [0.19-0.34] | 0.29 [0.22-0.38] | 0.28 [0.21-0.36] |
| **Outpatient consultation** |  |  |  |  |  |
| In treatment | 0.62 [0.02-3.48] | 1.19 [0.03-6.65] | 0.86 [0.02-4.78] | 0.62 [0.02-3.48] | 0.62 [0.02-3.48] |
| Post treatment | 0.51 [0.26-0.92] | 0.61 [0.20-1.43] | 0.57 [0.25-1.12] | 0.51 [0.26-0.92] | 0.47 [0.22-0.86] |
| **Residential rehabilitation** |  |  |  |  |  |
| In treatment | 0.00 [0.00-1.06] | 0.00 [0.00-3.16] | 0.00 [0.00-1.49] | 0.00 [0.00-1.06] | 0.00 [0.00-1.06] |
| Post treatment | 0.27 [0.11-0.56] | 0.60 [0.16-1.52] | 0.16 [0.03-0.47] | 0.27 [0.11-0.56] | 0.27 [0.11-0.56] |
| **Support and case management** | |  |  |  |  |
| In treatment | 0.07 [0.00-0.40] | 0.13 [0.00-0.73] | 0.09 [0.00-0.48] | 0.07 [0.00-0.40] | 0.07 [0.00-0.40] |
| Post treatment | 0.23 [0.14-0.35] | 0.27 [0.12-0.53] | 0.21 [0.12-0.34] | 0.23 [0.14-0.35] | 0.23 [0.14-0.35] |
| **Withdrawal management** |  |  |  |  |  |
| In treatment | 0.36 [0.01-1.99] | 0.00 [0.00-3.43] | 0.56 [0.01-3.15] | 0.36 [0.01-1.99] | 0.36 [0.01-1.99] |
| Post treatment | 0.28 [0.17-0.46] | 0.46 [0.21-0.87] | 0.16 [0.06-0.34] | 0.28 [0.17-0.46] | 0.28 [0.17-0.46] |

CI, confidence interval; CMR, crude mortality rate; N/A, not applicable; PY, person years.

## Table S5d. Results of sensitivity analyses – Opioids

|  | **CMR per 100 PY [95% CI]** | | | | |
| --- | --- | --- | --- | --- | --- |
|  | **Main analysis** | **2015-2018** | **Single services** | **0-day rule** | **7-day rule** |
| **Assessment only** |  |  |  |  |  |
| In treatment | N/A | N/A | N/A | N/A | N/A |
| Post treatment | 0.72 [0.47-1.07] | 1.00 [0.46-1.90] | 0.70 [0.45-1.04] | N/A | N/A |
| **Counselling** |  |  |  |  |  |
| In treatment | 0.43 [0.19-0.85] | 0.63 [0.20-1.46] | 0.41 [0.11-1.06] | 0.38 [0.15-0.77] | 0.54 [0.26-0.99] |
| Post treatment | 1.07 [0.85-1.33] | 1.51 [1.10-2.01] | 0.99 [0.72-1.31] | 1.08 [0.86-1.35] | 1.02 [0.80-1.27] |
| **Opioid agonist treatment** |  |  |  |  |  |
| In treatment | 0.84 [0.72-0.98] | 1.61 [1.27-2.01] | 0.85 [0.71-1.01] | 0.79 [0.67-0.92] | 0.95 [0.82-1.09] |
| Post treatment | 1.21 [1.07-1.37] | 1.63 [1.38-1.91] | 1.21 [1.06-1.38] | 1.25 [1.11-1.40] | 1.13 [1.00-1.28] |
| **Outpatient consultation** |  |  |  |  |  |
| In treatment | 1.09 [0.23-3.19] | 1.40 [0.17-5.07] | 1.88 [0.39-5.48] | 0.73 [0.09-2.63] | 2.18 [0.80-4.75] |
| Post treatment | 1.60 [1.08-2.29] | 1.82 [0.99-3.05] | 1.91 [1.23-2.85] | 1.66 [1.13-2.35] | 1.50 [0.99-2.16] |
| **Residential rehabilitation** |  |  |  |  |  |
| In treatment | 0.19 [0.00-1.03] | 0.00 [0.00-1.55] | 0.00 [0.00-0.93] | 0.19 [0.00-1.03] | 0.19 [0.00-1.03] |
| Post treatment | 0.79 [0.50-1.20] | 1.83 [1.04-2.97] | 0.69 [0.38-1.16] | 0.79 [0.50-1.20] | 0.79 [0.50-1.20] |
| **Support and case management** | |  |  |  |  |
| In treatment | 1.21 [0.66-2.03] | 2.81 [1.45-4.90] | 1.66 [0.86-2.90] | 1.03 [0.53-1.81] | 1.21 [0.66-2.03] |
| Post treatment | 0.72 [0.48-1.03] | 1.00 [0.55-1.68] | 0.69 [0.42-1.08] | 0.72 [0.48-1.03] | 0.70 [0.46-1.00] |
| **Withdrawal management** |  |  |  |  |  |
| In treatment | 1.08 [0.35-2.51] | 2.09 [0.25-7.56] | 0.89 [0.18-2.60] | 1.08 [0.35-2.51] | 1.94 [0.89-3.68] |
| Post treatment | 1.20 [0.94-1.52] | 1.94 [1.36-2.69] | 0.97 [0.68-1.34] | 1.25 [0.98-1.57] | 1.15 [0.89-1.46] |

CI, confidence interval; CMR, crude mortality rate; N/A, not applicable; PY, person years.

## Table S6. Age- and sex- standardised mortality rates for amphetamine-type stimulants and cannabinoids, per 100 PY [95% CI]

|  | **Amphetamine-type stimulants^a^** | **Cannabinoids** |
| --- | --- | --- |
| **Assessment only** |  |  |
| In treatment | N/A | N/A |
| Post treatment | 0.60 [0.07-1.13] | ^b^ |
| **Counselling** |  |  |
| In treatment | ^b^ | ^b^ |
| Post treatment | 0.93 [0.39-1.47] | 0.64 [0.36-0.91] |
| **Outpatient consultation** |  |  |
| In treatment | ^b^ | ^b^ |
| Post treatment | ^b^ | ^b^ |
| **Residential rehabilitation** |  |  |
| In treatment | ^b^ | ^b^ |
| Post treatment | ^b^ | ^b^ |
| **Support and case management** |  |  |
| In treatment | ^b^ | ^b^ |
| Post treatment | 1.23 [0.22-2.24] | 0.47 [0.00-1.07] |
| **Withdrawal management** |  |  |
| In treatment | ^b^ | ^b^ |
| Post treatment | 0.92 [0.14-1.70] | ^b^ |

CI, confidence interval; N/A, not applicable; PY, person years.

^a^ For people with amphetamine-type stimulants as the principal drug of concern, the 55-64 years age group was combined with the ≥ 65 years group to overcome small cell limitations. ^b^ standardised mortality rate not estimable because total deaths <20 and/or number of people in at least one age-sex stratum <20.
